# Automated Counting of Rice Panicle by Applying Deep Learning Model to Images from Unmanned Aerial Vehicle Platform

**DOI:** 10.3390/s19143106

**Published:** 2019-07-13

**Authors:** Chengquan Zhou, Hongbao Ye, Jun Hu, Xiaoyan Shi, Shan Hua, Jibo Yue, Zhifu Xu, Guijun Yang

**Affiliations:** 1 Institute of Agricultural Equipment, Zhejiang Academy of Agricultural Sciences (ZAAS), Hangzhou 310000, China; 2 Key Laboratory of Quantitative Remote Sensing in Agriculture of Ministry of Agriculture P. R. China, Beijing Research Center for Information Technology in Agriculture, Beijing 100089, China; 3 Key Laboratory of Agri-informatics, Ministry of Agriculture, Beijing 100089, China

**Keywords:** rice panicle counting, UAV platform, deep learning, yield estimation

## Abstract

The number of panicles per unit area is a common indicator of rice yield and is of great significance to yield estimation, breeding, and phenotype analysis. Traditional counting methods have various drawbacks, such as long delay times and high subjectivity, and they are easily perturbed by noise. To improve the accuracy of rice detection and counting in the field, we developed and implemented a panicle detection and counting system that is based on improved region-based fully convolutional networks, and we use the system to automate rice-phenotype measurements. The field experiments were conducted in target areas to train and test the system and used a rotor light unmanned aerial vehicle equipped with a high-definition RGB camera to collect images. The trained model achieved a precision of 0.868 on a held-out test set, which demonstrates the feasibility of this approach. The algorithm can deal with the irregular edge of the rice panicle, the significantly different appearance between the different varieties and growing periods, the interference due to color overlapping between panicle and leaves, and the variations in illumination intensity and shading effects in the field. The result is more accurate and efficient recognition of rice-panicles, which facilitates rice breeding. Overall, the approach of training deep learning models on increasingly large and publicly available image datasets presents a clear path toward smartphone-assisted crop disease diagnosis on a global scale.

## 1. Introduction

Rice is recognized as the most significant crop species worldwide, and the current annual production of rice grain is 590 million tons [1]. High yield has always been one of the most important objectives of rice breeding and cultivation. Rice breeding requires measuring the yield of a large number of candidate samples in different environments, so as to provide a basis for breeding high-yield, high-quality, stress-resistant rice varieties. The rice panicle is the organ for the growth of rice grains and is directly related to final yield. It also plays an important role in pest detection, nutritional diagnosis, and growth-period detection [2]. Therefore, the accurate recognition of rice panicles is a key step to obtain panicle characteristics and to automate the detection of rice phenotypes. The appearance of panicles—such as shape, color, size, texture, and posture—vary strongly among the different rice varieties and growth stages. The edge of the rice panicle is very irregular, and the panicle color blends with that of the leaves. The complex field environment, mutual occlusion between different rice organs, and uneven and constantly changing natural solar illumination severely hinder efforts to automatically recognize rice panicles [3]. Early work on recognizing plant organs from images focused on indoor experiments and used ground-based vehicles to collect data. An example of such work is Phadikar et al., who describe an image-based system to detect rice disease [4]. They used a digital camera to image the infected plants and processed the images by using image-growing and segmentation techniques. They then used a neural network to separate infected leaves from normal leaves and thereby determined the number of diseased plants. In other work, Huang et al. measured the panicle length by using a dual-camera system equipped with a long-focus lens and a short-focus lens [5]. After co-registration and resampling a series of images, the panicle length was calculated as the sum of the distances between each adjacent point on the path. More recently, Fernandezgallego et al. proposed a novel ear-counting algorithm to measure ear density under field conditions [6]. The whole algorithm contained three steps: (i) remove low- and high-frequency elements appearing in an image by using a Laplacian frequency filter, (ii) reduce noise by using a median filter, and (iii) segment the images. After these treatments, they could accurately deduce the number of ears with high efficiency. However, these color-based or machine-learning-based methods are susceptible to illumination and other factors [7]. They are thus only suitable for a specific growth period and are very sensitive to environmental noise.

In recent years, to address a number of plant-phenotyping problems, deep learning (DL) has been adopted as the method of choice for many image analysis applications [8]. DL networks avoid the need for hand-engineered features by automatically learning feature representations that discriminately capture the data distribution. The hidden layer of the network extracts features, and the feature information is reflected in the weight of the hidden-layer links. The parallel structure of neural networks makes them insensitive to incomplete input mode information or defective features [9]. One such study organized by Pound et al. recognized the characteristic parts of wheat by using a convolutional neural network (CNN) technique. Xiong et al. introduced a panicle-SEG algorithm based on super-pixel segmentation and a CNN [10]. The method involves several steps, including super-pixel region generation, convolutional neural network classification, and entropy rate super-pixel optimization. After training the model by using 684 manual labeling images, the average precision of panicle-SEG reached 82%, which shows its reliability. In addition, Rahnemoonfar et al. used DL to accurately count the number of fruit in an unstructured environment. Their research included a fully convolutional network to extract candidate regions and a counting algorithm based on a second convolutional network to estimate the number of fruit in each region [11]. Most research that incorporated DL architectures took advantage of transfer learning, which concerns leveraging the already existing knowledge of some related task or domain to increase the learning efficiency of the problem under study by fine-tuning pre-trained models [12]. The considerable drawbacks of this algorithm or model are (1) the limitation of image data acquisition, and (2) the requirement of large datasets.

Monitoring rice panicles by using images acquired from rotor light unmanned aerial vehicles (RL-UAVs) in the field is complicated by the following difficulties:

- The shape, color, size, texture, and posture of panicles of different varieties and growth stages differ significantly. The panicle edge is very irregular, and the panicle color is similar to that of leaves.
- The natural environment is complex and includes mutual occlusion of different rice organs and varying soil reflectance and light intensity.
- Restrictions of RL-UAV flight altitude and sensor resolution affect the accuracy of automated identification and manual labeling, which can lead to false positives and false negatives.

This paper proposes a method to detect rice panicles based on statistical treatment of digital images acquired from a UAV platform. The aim of this study is to train and apply a CNN-based model called the ‘improved region-based fully convolutional network’ (R-FCN), which can accurately count rice panicles in the field. The training process produces a series of bounding boxes that contain detected panicles and compares the number of panicles in each box with manual labeling results to determine the effectiveness of the proposed method. Moreover, we dynamically monitor the number of panicles of different rice varieties at different growth stages and analyze the error cases and limitations of the proposed DL-based model. The proposed method provides automated panicle recognition with the accuracies >90% in real time, which is superior to the results of several traditional training models. The improved R-FCN method offers the advantage of object recognition from image sets, which allows it to perform better than the other methods.

The outline of this article is as follows: The Materials and Methods section describes the study area and the image-acquisition system. We also present the image-dataset preprocessing process. Finally, several DL models are introduced, following which we describe in detail the proposed improved R-FCN method to monitor rice panicles. The Results and Discussion section analyzes the accuracy of each model by using four evaluation indexes. We also analyze the number of panicles at different growth stages. Finally, the last section summarizes the works presented in this paper and presents the main conclusions.

## 2. Materials and Methods

### 2.1. Study Area

This experiment was conducted from June to October at the Agricultural Genomics Institute at Shenzhen, Chinese Academy of Agricultural Sciences (AGIS-CAAS) Research Base, Shenzhen City, Guangdong Province, China (Latitude 22°60′N, longitude 114°51′E). Shenzhen City has an average temperature of 24.0 °C and an average rainfall of 1933.3 mm (acquired from China Meteorological Data Service, http://data.cma.cn/). The chemical properties of the experimental area are listed in Table 1.

### 2.2. Field Experiments and Image Acquisition

For this study, we planted three rice hybrids labeled XH34, XH40, and XH166 in three plots. The plots were 3 m wide, with 8-m-long rows with an inter-row spacing of approximately 0.5 m, with a gap of approximately 0.7 m between plot rows and 0.3 m between columns. The planting density in each plot was 75 plants/m^2^. Each plot was imaged once a week from 1 September to 20 October, 2018. The image datasets were captured by using a commercial six-rotor UAV (DJI M-600, DJI Corporation, Shenzhen, China) equipped with an external digital camera (Sony QX-100, Sony Corporation, Tokyo, Japan) with a 1 inch Exmor R CMOS and a 60° field of view. All images were captured from a height of 17 m above the ground and stored in JPEG format for preprocessing. The images for this work were acquired between 10:00 a.m. and 12:00 p.m. to avoid overexposure and to ensure uniform lighting while the UAV flew over the plots. The UAV system flew 17 m above the ground to ensure that the panicles could be clearly identified. The remaining camera settings were as follows:

• Focal length8.8 mm• ISOAutomatic• Exposure time1/90 s• Aperturef/3.9• No. of pixels5472 × 3048

**Figure 1:**
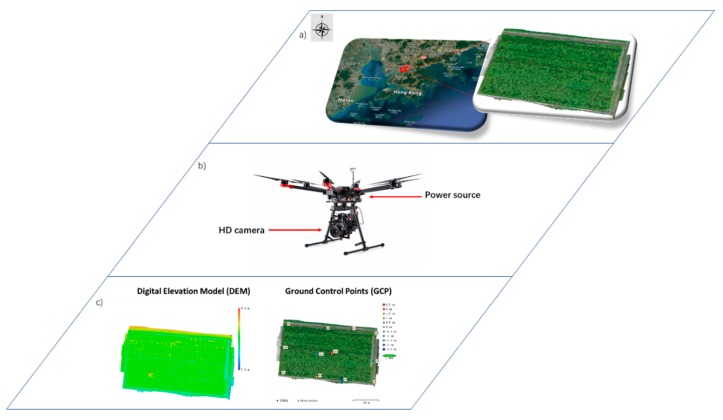
(**a**) Location of experimental plot, (**b**) unmanned aerial vehicle platform, and (**c**) ground control points, and ground control points in acquired data.

To satisfy the requirement of 50% side overlap and 70% longitudinal overlap, the UAV was flown at 5 m/s. Moreover, it had six POS types: longitude, latitude, height, pitch, roll, and yaw. The final rice panicle image dataset consisted of 235 images with a ground resolution of 2.51 mm/pixel (Figure 1).

### 2.3. Data Preprocessing

**Figure 2:**
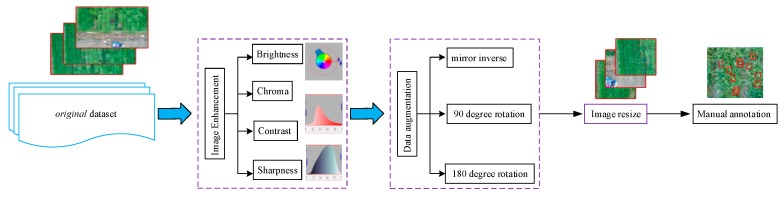
Flowchart showing data-preprocessing procedure.

The high-quality UAV-based images were used to construct an original dataset, which is a key contribution of this study. We then divided the image preprocessing pipeline into four main steps: First, the image-enhancement process was used to eliminate the effect caused by shadow and variable illumination and to remove the background. Next, the downscale images were used to adapt the DL model and improve training efficiency. We then added annotations to each image denoting the bounding boxes of the panicles. Finally, we used data-augmentation techniques to artificially enlarge the number of training images [13] (Figure 2).

#### 2.3.1. Image Enhancement and Data Augmentation

Because of the uncertainty in the direction of sunlight and weather, the field illumination condition can be very complex. To enrich the image training set and avoid overfitting [14], we enhanced the dataset. Image enhancement serves to eliminate the illumination effect and allows the foreground of the image to be clearly identified. In this study, to facilitate future generalization of the training model, the original images were processed by using eight different methods: (a) brightness enhancement and attenuation, (b) chroma enhancement and attenuation, (c) contrast enhancement and attenuation, and (d) sharpness enhancement and attenuation. The settings about the image enhancement process are given in Table 2.

After applying such processing to the original dataset, the images are enhanced by removing the effect of shadow or change of illumination intensity, which will improve the accuracy of the proposed system [15]. 

Data augmentation is a common way to expand the variability of training data by artificially enlarging a dataset via label-preserving transformations. Typical augmentation techniques used in the computer vision community include left-right flipping, image re-scaling, and changing image color. In this study, we use three techniques to augment the dataset: (1) mirror inverse, (2) 90° rotation, and (3) 180° rotation. These operations are implemented through a Python script.

#### 2.3.2. Image Resize

All the images were resized to adapt to the requirement of the training model [16]. Currently, the sizes used in this step are 256 × 256, 128 × 128, 96 × 96, and 60 × 60 pixels in common. In this study, we resized the images to 256 × 256 to match the size of the rice panicle, and we performed all our training and validation on these processed images.

#### 2.3.3. Manual Annotation

The ground truth for panicles was collected by using several rectangular annotations. For the annotation of images, three experts labeled the resized images by using the publicly available tool *LabelImg* (acquired from https://github.com/tzutalin/labelImg). Each labeled image has an additional XML file containing the coordinates of the annotated bounding boxes. Each box is represented as a four-dimensional array (x_min_, y_min_, x_max_, y_max_) to determine its relative position on the graph. After this treatment, each image contains about 800–1000 boxes that represent the corresponding number of panicles.

### 2.4. Deep Learning Architecture

With the development of DL theory, many deep neural network models have been proposed, including AlexNet, ZF, VGG, GoogleNet, and ResNet [17]. Each of these networks can build network models of different depths by designing different weighting layers. Although deeper networks may bring higher accuracy, they will also reduce the speed of training and detection. In this paper, we focus on three popular architectures: AlexNet, VggNet, Inception-V3, and an improved R-FCN model.

#### 2.4.1. AlexNet

The original AlexNet was proposed by Hinton and Alex Krizhevsky when they competed in the ImageNet Competition in 2012 [18]. AlexNet consists of five convolution layers, five pooling layers, three full connection layers, and a Softmax layer that can maximize the target value of logistic regression for classification. The architecture can maximize the average logarithmic probability of correct labels in training samples under distributed conditions. Here, we use the ReLU activation function rather than the Sigmoid function to more rapidly solve the gradient dispersion problem caused by the deep network. The dropout layer can effectively reduce the overfitting phenomenon. The overlapping maximum pooling layer avoids the fuzzification effect of the average pooling layer, and the step size is smaller than that of the pooling core. Therefore, there is overlap and coverage between the pooling output layers, which improves the richness of the features. Local response normalization is used to create competition for local neuron activity, which further increases the larger response and restrains the smaller feedback neurons, thus facilitating the future generalization of the model.

#### 2.4.2. VggNet

VggNet is the extension of the AlexNet model [19] and consists of 5 maximum pooling layers, 13 convolution layers, 3 full connection layers, and a SoftMax Classifier Layer. The convolution kernels are 3 × 3, which allows for better extraction of image details and enhances the nonlinear expression of the network (stride = 1). The max pooling technique is used in all pooling layer, and the size of the pooling window is 2 × 2 (stride = 2). All hidden layers are added with ReLU layers. After the first and second full connection layers, the dropout technology is also used to prevent network overfitting. To reduce memory consumption and computing time, VggNet does not use local response normalization. 

#### 2.4.3. Inception-V3

Inception-V3 model [20] has 13 layers, including 6 convolution layers, 2 pooling layers, 3 inception layers, 1 full connection layer, and 1 soft max output layer. Among them, the convolution core size of the C_1_ and C_5_ layers is 3 × 3, the convolution slip value is two, the size of the C_2_, C_3_, C_4_, and C_6_ layers is 3 × 3, and the convolution slip value is 1. The idea of the inception layer is to stack convolution cores of different convolution window sizes. It can increase the width of the network and the adaptability of the network to scale.

#### 2.4.4. Improved R-FCN

In this paper, we proposed an improved panicle detection model based on Region-based Fully Convolutional Networks (R-FCN) [21]. To make the model training more complete, the online hard example mining method is used to relax the constraints of positive and negative samples, which extends the scope of the training set. For the overlapping problem, a linear non-maxima suppression method is used to avoid missing detection. Target detection based on the R-FCN model mainly consists of two steps: (1) extracting regions of interest from the shared characteristic map by region proposal networks, and (2) the regions of interest of subnet are used as classification network to classify and recognize regions of interest. 

Combining with the specific task of rice panicle detection, an improved R-FCN model is proposed in this paper. The improved network has two highlights:

- Online hard example mining was used to identify some more-complex targets. Online hard example mining calculates the loss of candidate regions provided by region proposal networks, ranks these candidate regions according to the value, and selects *K* target regions with the largest loss value as the hard example to join the net for training.
- When the R-FCN model receives the detection frame, the non-maximum suppression algorithm is used to obtain the optimal coordinates of the target accurately and remove the duplicate boundary frames. Non-maximum suppression can be expressed by the fractional reset function

(1) $$s_{i}=\{\begin{array}{c} s_{i},iou(M,b_{i})<N_{t}\\ 0,iou(M,b_{i})\geq N_{t} \end{array}$$

where *s_i_* is the confidence of the detection frame, *M* is the location of detection frame with highest confidence, *b_i_* is the location of the detection frame, *N_t_* is the overlap threshold, and *iou* (*M*, *b_i_*) is the overlap rate of *M* and *b_i_*. The non-maximum suppression threshold parameter was also optimized over the validation set and ranged from 0.2 to 0.4 for the different fruits; however, the results were not sensitive in this range.

### 2.5. Transfer Learning

We now evaluate how the four DL architectures above perform on the dataset by training the model from scratch in one case and then by adapting already trained models (on the ImageNet dataset) by using transfer learning. The transfer learning uses a large basic network to train the CNN, and then transfers the learned functions to a new target. The ImageNet dataset is used to pre-trained CNN features, and state-of-the-art results have been obtained on a variety of image-processing tasks, ranging from image classification to image captioning. For transfer learning, we re-initialize the weights of layer fc8 in case of AlexNet, add two full connection layers in VggNet with the number of nodes 2048 and 11, add one full connection layer with a 1024 one-dimensional vector input and an output layer of 8-bit soft Max classifier in the feature extraction part of Inception-V3. The hyper-parameters for all experiments are given in Table 3.

To test the accuracy of the model processing for unknown data and whether it can effectively prevent overfitting, we cut the whole dataset into different proportions: 80-20 (80% of the whole dataset used for training, and 20% for testing), 60-40 (60% of the whole dataset used for training, and 40% for testing), 50-50 (50% of the whole dataset used for training, and 50% for testing), 40-60 (40% of the whole dataset used for training, and 60% for testing), and 20-80 (20% of the whole dataset used for training, and 80% for testing) [22].

### 2.6. Evaluation Index

The output of the study is a list of bounding boxes that contain all the panicles. The accuracy of these models was then evaluated by using three quality factors: mean precision, mean recall, and mean F-measure [23]. Mean precision reflects the accuracy of the model, and mean recall represents the completeness of the captured panicles. In practice, mean precision and mean recall interact with each other. When mean precision is high, mean recall is low. Furthermore, F-measure is proposed to balance the other two indicators. The higher the value of the F-measure, the more perfect the final results will be. The formulas for these evaluation indicators are

(2) $$Precision=\frac{TP}{TP+FP}$$

(3) $$Recall=\frac{TP}{TP+FN}$$

(4) $$F=\frac{2\times Precision\times Recall}{Precision+Recall}\%$$

where true positive (*TP*) in Equations (2) and (3) represents the correct classification of a region as a panicle, false positive (*FP*) represents the incorrect classification of a background region as a panicle and multiple detections of the same panicle. False negative (*FN*) indicates an incorrect classification of a panicle as a background region. 

In addition, the mean intersection over union (*mIOU*) was used to evaluate processing precision on the validation sets. It generates two boxes called ‘predicted bounding box’ and ‘ground-truth bounding box’ and then compares the rate of overlap between them. The schematic diagram and computational formula of the *mIOU* are

(5) $$mIOU=mean(\frac{DetectionLeafArea\cap GroundTruth}{DetectionLeafArea\cup GroundTruth})$$

Our goal is to take the training images plus bounding boxes, construct an object detector, and then evaluate its performance on the testing set. The *mIOU* changes within the interval [0,1] and a *mIOU* score greater than 0.5 is normally considered a ‘good’ prediction. We treat a detection task as positive if the *mIOU* value between the predicted and the ground truth bounding box is greater than 0.2. This equates to a 58% overlap along each axis of the object, which was considered sufficient for counting panicles.

## 3. Results and Discussion

The experiment was run on the Ubuntu 16.04 operating system (OMEN by HP Laptop with a 4-core i5 CPU, 2.3 GHz per CPU core, 8 GB of memory, and an NVIDA GTX 960M video card). Based on Cafe’s deep-learning framework, we use Python to do the training and testing of the target-recognition network model. In this paper, we use the stochastic gradient descent method to train the network in a joint end-to-end manner. The pre-training model on ImageNet is used to initialize the network parameters, and the verification period is set to 1000 (i.e., the accuracy of the training model is tested 1000 times on the verification set per iteration of the network).

### 3.1. Performance

**Figure 3:**
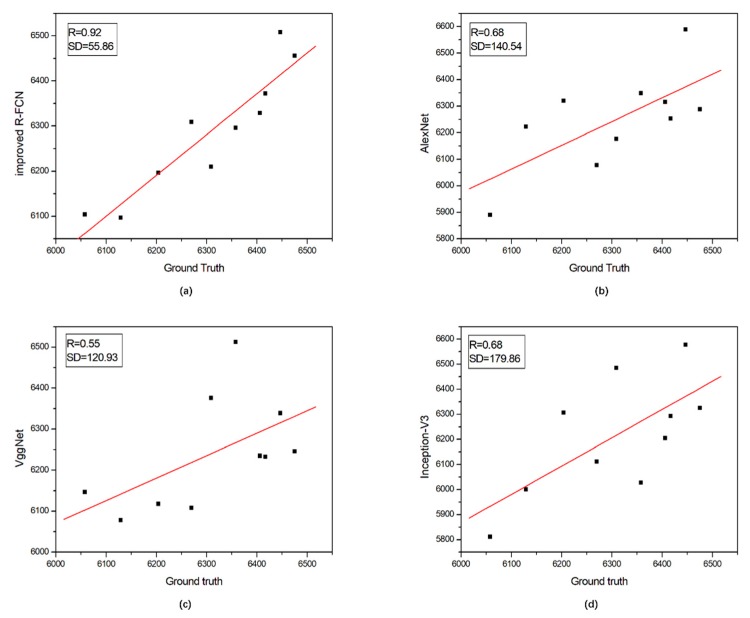
Ground truth versus estimated number of panicles per plot. (**a**) represents the correlation between the ground truth and improved R-FCN, (**b**) represents the correlation between the ground truth and AlexNet, (**c**) represents the correlation between the ground truth and VggNet and (**d**) represents the correlation between the ground truth and Inception-V3. The vertical axis refers to the number of panicles estimated by the proposed approach and the horizontal axis refers to the number of panicles that have been manually counted.

After dealing with the images in the testing set, the four models above returned the location of the detected panicles and their total number; see Figure 3 (10 randomly selected plots). For each combination of proportions, the following statistics are provided in Table 4: mean precision, mean recall, F-measure, and *mIOU*. Across all our experimental configurations, the overall precision obtained on the dataset varied from 0.592 (for 20–80 AlexNet) to 0.897 (for 80–20 improved R-FCN), which shows the strong potential of DL technology for such recognition problems. Specifically, AlexNet has relatively low precision (mean value 0.651); VggNet and Inception-V3 are second and third with average precisions of 0.802 and 0.818 and a much higher recall of 0.814 and 0.833, respectively. Of all these methods, the improved R-FCN method gives the highest mean precision (0.868), the highest recall (0.883), and the highest F-measure (0.874). For the *mIOU* index, the VggNet has the highest rate of misclassified pixels (0.792) whereas the improved R-FCN method performs best with the lowest rate of misclassified pixels (0.887). To address the issue of overfitting, different test set to train set ratio were used to cut the whole dataset even in the extreme case: 20% of the whole dataset used for training, and 80% for testing. As expected, these four models will perform worse if we continue to increase the ratio of testing set to training set, but if the model indeed overfits, then the drop-in performance is not as dramatic as we expected (Figure 3). 

In addition to detection accuracy, another important performance index of target detection algorithm is speed. Only with high speed can real-time detection be achieved, which is extremely important for some application scenarios. Average running time (ART) represents the time taken by different models to process a certain picture on the same hardware, and the shorter the time, the faster the speed. Since the steps of image acquisition and preprocessing are the same, we here compared the training efficiency of different methods and demonstrated the results in Table 5. 

**Figure 4:**
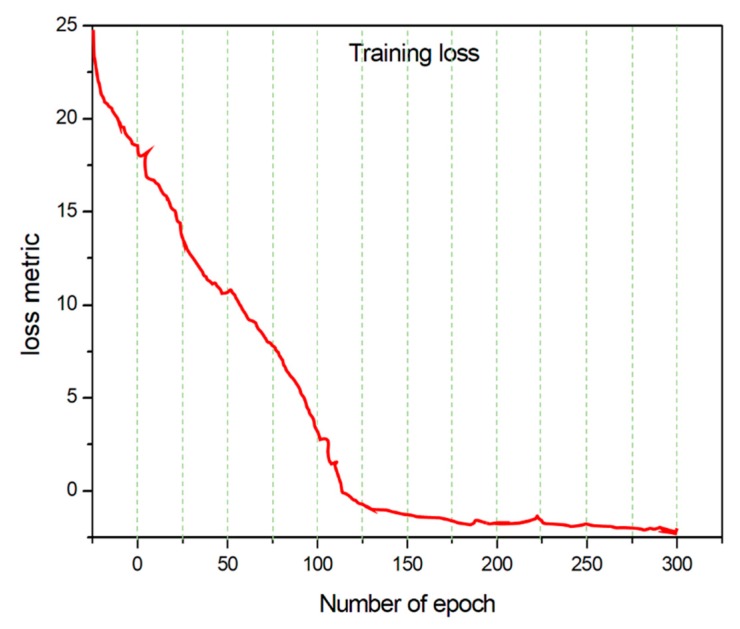
Number of epochs versus training loss (e.g., 300 rounds).

From Table 5, one can see that the time required for panicle detection is always within the range of 0.5 s for all tested images. This is a quite satisfactory result after considering its future application scenarios for miniaturized devices. Also, the training loss and error rates while the model is learning can be used to judge the efficiency of a training model. We define herein the epoch as one full pass forward and backward through the network during the learning stage. After a series of epochs, the weights of the model will become closer to a suitable range and will reduce the error rate and training loss. Figure 4 shows the relationship between the loss metric and the number of epochs. We see from Figure 4 that the loss metric decreases over subsequent epochs of training and the loss and error rate remain almost unchanged after 100 epochs. To avoid overfitting, the number of epochs is fixed to 300 to achieve a relatively accurate result (Figure 4).

The accuracy of the proposed model differs substantially for changes in training images, transfer learning, and data augmentation. Here, we analyze how these factors affect the accuracy of the recognition results.

A. Number of Training Images

**Figure 5:**
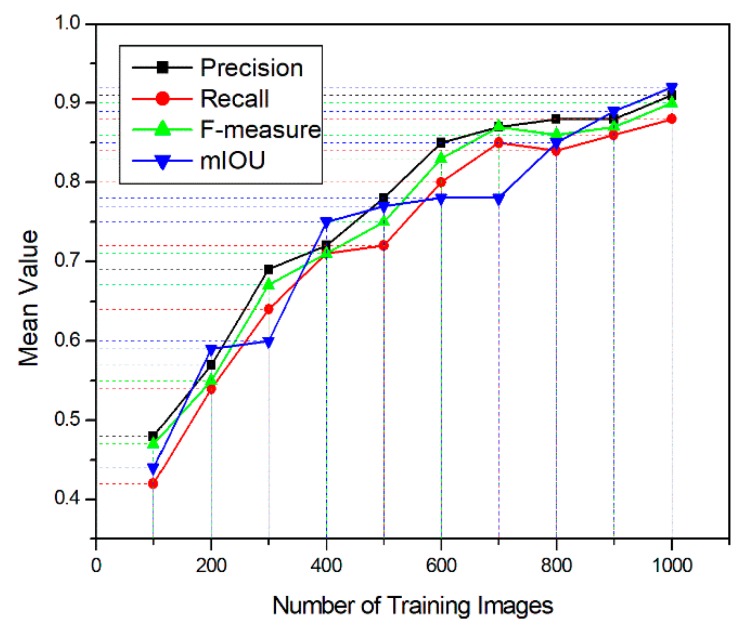
Detection performance for panicles in terms of average evaluation index value versus number of training images.

Figure 5 shows the relationship between evaluation index and the number of training images. We see from Figure 5 that the index rises quickly with a small number of training images. For example, when the accuracy reaches a relatively high value (about 0.85), performance is close to convergence for all models, only increasing by 0.05 to 0.07 after introducing the remaining training images (Figure 5). 

B. Transfer Learning

**Figure 6:**
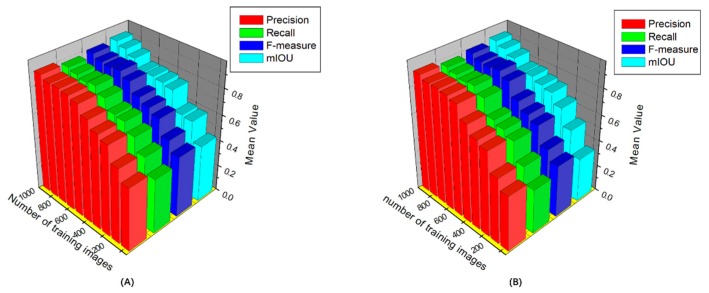
Detection performance with and without transfer learning for different numbers of trainings: (**A**) with transfer learning, (**B**) without transfer learning.

Transfer learning involves the migration of the original data domain and the original task to the target data domain and target task, respectively. The weight parameters are used to improve the prediction function for the target domain. In this study, most parameters in the training models were directly initialized from ImageNet. To examine the utility of transfer learning, we compare the accuracy of the proposed model by using pre-trained models from ImageNet and by training from scratch. The results reveal a certain difference in initial accuracy (from 5% to 15%). After a series of image training processes, the initial benefits diminish, with a difference of less than 1% remaining between the highest and the lowest accuracy (Figure 6). 

C. Data Augmentation

**Figure 7:**
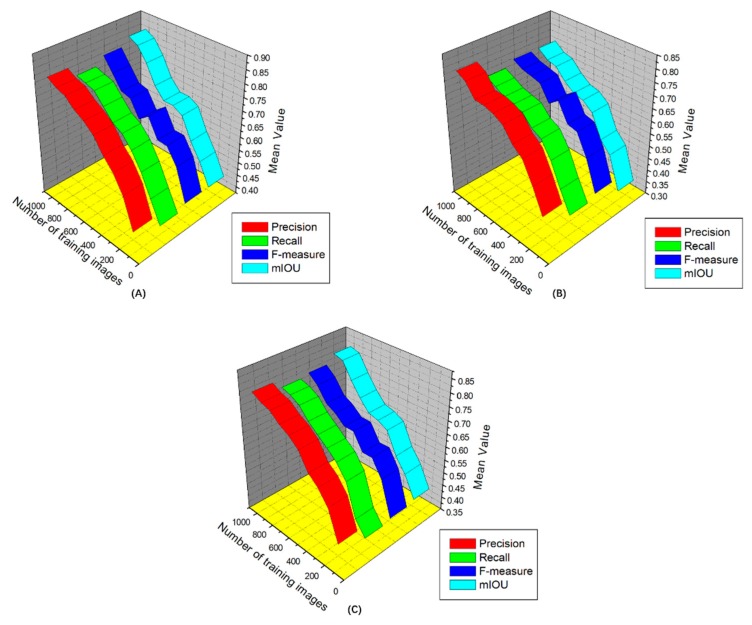
Detection performance for different number of training instances with different data augmentation procedures used during training: (**A**) training with original dataset, (**B**) using color transformation, (**C**) using shape and scale variability.

The aim of the data augmentation is to enlarge the number of training images, which can improve the overall learning procedure and performance. This process is important for DL trainings that possess small datasets and is especially important when training models by using synthetic images and testing them on real images. By augmenting the training set, the model can more easily be generalized to adapt to real-world problems. We see from Figure 7 that, after various augmentations, the model becomes significantly more robust. Specifically, the color transformation improves the final accuracy by about 10%, and the mirror and rotation processes improve by 5% to 7% compared with non-augmented data. These results suggest that there are more color variations in the dataset relative to shape and scale variability (Figure 7). 

### 3.2. Dynamic Monitoring of Panicle Number at Different Growth Stages

Rice yield can be decomposed into four elements: panicles per unit area, total grains per panicle, setting percentage, and grain weight. The number of panicles per unit area is the most active and basic factor in yield components. To dynamically monitor rice panicle number during the different growth stages, the panicles per unit area of three varieties of rice grown under different fertilizer treatments (called no treatment, early treatment, and late treatment) were compared and analyzed. Two-thirds of the plots were treated at a standard rate of 139.5 kg/hm^2^ N, 57.75 kg/hm^2^ P_2_O_5_ and 120.0 kg/hm^2^ K_2_O while the rest plots received no treatment. For early treatment, the fertilizers were applied on 24 June, 7 July, 21 July and 11 August, respectively. For the late stage treatment, all fertilizers were applied together on 15 July. To verify that panicles are correctly detected and counted, we systematically tested 150 rice images (50 images per treatment plan). To reduce the effect of panicle overlap on counting accuracy, the thresholds of panicle confidence fraction *P* and overlap-area ratio *I* were selected through several experiments. Finally, *P* and *I* were set to 0.95 and 0.1, respectively. The measured data of rice panicle in the image were obtained by manual counting. Because of the incomplete situation in the region of the panicle edge, the automated count is greater than the manual count. To ensure the accuracy of the count and the rigor of the experiment, we applied a unified principle of rice panicle counting: Five experts separately counted the panicles in each image. For each image, the average manual count is used as the number of panicles in the image to reduce the subjective error of automated counting. Table 6 gives both the manual counts and the automated counts.

We see from Table 6 that the untreated plants generally produced fewer panicles per square meter than the plants that had undergone either the early or late treatment. Each variety of rice shows a significant difference in panicle number between no treatment and treatment, which demonstrates that they were sensitive to the amount of nitrogen.

For nearly all rice varieties, significantly higher yields were obtained for early treatment than for late treatment. These results also show that the rice yield is quadratic in nitrogen uptake and in nitrogen application rate. With the increasing application of nitrogen, plant nutrient content, and nutrient uptake increase continuously. At present, few studies have concentrated on the characteristics of nitrogen uptake and utilization in rice under the condition of precise nitrogen application. In the future, the relationship between nitrogen-application time and amount, and rice biomass needs to be further studied. 

### 3.3. Error Case and Limitation

Target detection is an important subject in the field of computer vision. The main task is to locate the object of interest in an image. Doing so accurately determine the specific category of each object and give the boundary frame of them. However, since these methods use designed features, even the best nonlinear classifier is unable to make the right judgment. The performance of convolutional neural networks in object recognition and image classification has made tremendous progress in the past few years. Using the deep convolutional neural network architecture, we trained a model on the images of rice canopy with the goal of identifying the panicle part and counting the number of it. However, there are a number of error cases and limitations at the current stage that need to be addressed in future work. 

#### 3.3.1. Error Cases

A portion of the observed detection errors can be attributed to (1) the deep-learning results in a black-box model, which are almost impossible to explain based on the technical and logical basis of the system, (2) a lack of high-quality labeled training samples leads to overfitting and poor model robustness, (3) the inability to detect overlapping panicles in an image, and (4) errors in manual labeling, which leads to errors in validation. 

#### 3.3.2. Limitations

Deep learning focuses mainly on CNNs in the image field, but the convolution operation requires the entire network to have a large amount of computation so that network training takes an excessively long time. Changing the form of the convolution operation to simplify the computational complexity should be a major development direction. A second limitation is that we are currently constrained to the classification of single panicles, facing up, on a homogeneous background. Although these are straightforward conditions, a real-world application should be able to classify images of panicles as presented directly on the plant. At present, experiments are used to prove the effectiveness of CNNs. The training parameters are based mostly on experience and a lack of theoretical guidance and quantitative analysis. A more reasonable network structure should be designed for target detection and to improve detection efficiency by combining recurrent neural networks to achieve multi-scale and multi-category target detection.

## 4. Conclusions

Estimating the number of rice panicles in the field is a challenging task. However, it is an important index for plant breeders in order to select high-yield varieties. Most previous work involving the analysis of panicle images was done in a controlled environment and was not suitable for practical applications. In this paper, we present a panicle detection system based on a state-of-art DL model applied to images acquired from a UAV platform. The model can accurately detect rice panicles in images acquired in a complex and changing outdoor environment. The analysis of transfer learning shows that transferring weights between orchards does not lead to significant performance gains over a network initialized directly from the highly generalized ImageNet features. Data augmentation techniques, such as flip and scale improve performance, giving equivalent performance with less than half the number of training images. The model produced an average precision, recall, F-measure, and *mIOU* of 0.868, 0.883, 0.874, and 0.887 respectively, which indicates that it is very precise. Also, the high ART of less than 0.5 s shows the great application potential of our model, trained on UAV image dataset. The main contributions of this paper is (1) the design of an improved R-FCN model that not only accurately recognizes panicles, but also simplifies and accelerates network training, and (2) a model that accurately recognizes incomplete or small panicles in images and that can be used in real time. Although the results of this method are satisfactory, the model remains too large, and the training process needs to be improved. A more powerful central processing unit and graphics processors unit, such as new products of the Xeon series and NVIDIA GeForce, will greatly reduce the training time of our model. In the future, panicle detection will be further analyzed to better understand transfer learning between datasets representing a single rice variety but captured under different lighting conditions, sensor configurations, and seasons.

## Figures and Tables

**Table 1:** Chemical properties of experimental area.

Organic Matter	Nitrogen (N)	Phosphorus (P)	Potassium (K)	pH
30.6 g·kg^−1^	1.56 g·kg^−1^	35.2 mg·kg^−1^	89 mg·kg^−1^	7.4

**Table 2:** Parameter adjustments.

Parameter	Enhancement (%)	Attenuation (%)
Brightness	120	60
Chroma	120	60
Contrast	120	60
Sharpness	200	10

**Table 3:** Hyper-parameters for all experiments.

Hyper-Parameter	AlexNet	VggNet	Inception-V3	Improved R-FCN
Base learning rate	0.0005	0.001	0.01	0.01
Momentum	0.9	0.9	0.9	0.9
Dropout	0.5	0.5	0.5	0.5
Batch size	64	40	100	100
Iteration times	5000	5000	5000	5000

**Table 4:** Evaluation and validation of panicle detection using different models applied to UAV image dataset. ‘1’ represents AlexNet, ‘2’ represents VggNet, ‘3’ represents Inception-V3, and ‘4’ represents improved R-FCN.

Methods	Proportion Combination	Precision	Recall	F-Measure	mIOU
1	80-20	0.731	0.699	0.711	0.821
1	60-40	0.684	0.672	0.681	0.808
1	50-50	0.626	0.615	0.619	0.810
1	40-60	0.622	0.596	0.603	0.796
1	20-80	0.592	0.583	0.590	0.784
2	80-20	0.819	0.819	0.807	0.803
2	60-40	0.821	0.831	0.828	0.796
2	50-50	0.805	0.825	0.817	0.793
2	40-60	0.792	0.808	0.801	0.785
2	20-80	0.773	0.787	0.781	0.783
3	80-20	0.834	0.879	0.854	0.799
3	60-40	0.835	0.871	0.862	0.804
3	50-50	0.827	0.824	0.820	0.851
3	40-60	0.801	0.806	0.805	0.846
3	20-80	0.793	0.785	0.791	0.831
4	80-20	0.866	0.904	0.896	0.925
4	60-40	0.897	0.901	0.891	0.892
4	50-50	0.872	0.897	0.875	0.889
4	40-60	0.861	0.870	0.865	0.886
4	20-80	0.844	0.843	0.843	0.843

**Table 5:** ART of different methods. ‘1’ represents AlexNet, ‘2’ represents VggNet, ‘3’ represents Inception-V3, and ‘4’ represents improved R-FCN.

Methods	ART/s
1	0.482
2	0.573
3	0.496
4	0.489

**Table 6:** Count of rice panicles for different types of treatments.

Rice Varieties	No Treatment									
	1	2	3	4	5	6	7	8	9	10
XH34	5217	5563	5218	5119	5534	5168	5197	5984	6107	5547
XH40	5718	5213	5549	5769	5421	5533	5697	6078	6124	6057
XH166	5533	5478	5961	5311	5426	5697	5321	5514	5321	5578
	**Early Treatment**									
XH34	6210	6587	6412	6622	6478	6271	6978	6389	6584	6397
XH40	6648	6913	6875	6389	6472	6196	6389	6145	6089	6552
XH166	6315	6298	6103	6917	6122	6308	6102	6301	6559	6789
	**Late Treatment**									
XH34	6987	6359	6555	6471	6216	6333	6987	6874	6222	6315
XH40	6321	6975	7004	6894	7105	7056	7059	6981	6781	6145
XH166	6326	6521	6895	6987	7582	7002	6798	6903	6711	6579
